# Supplementary material for: Dynamic core-periphery structure of information sharing networks in entorhinal cortex and hippocampus
Source: Netw Neurosci. 2020 Sep 1;4(3):946–75. doi: 10.1162/netn_a_00142 (PMC7888487; doi:10.1162/netn_a_00142)
Supplement: Supplementary file 1 [file netn-04-946-s001.pdf]

Pedreschi, N., Clawson, W., Bernard, C., Quilichini, P., Barrat, A. & Battaglia, D. (2020). Supporting information for "Dynamic core periphery structure of information sharing networks in entorhinal cortex and hippocampus." *Network Neuroscience 4*(3), 946-975. https://doi.org/10.1162/netn_a_00142

Dynamic core periphery structure of information sharing networks in entorhinal cortex and hippocampus

Pedreschi, N.[1,2], Clawson, W.[2], Bernard, C.[2], Quilichini, P.[2], Barrat, A.[1,3], and Battaglia, D.[2]

[1]Aix Marseille Univ, Université de Toulon, CNRS, CPT, Turing Center for Living Systems, Marseille, France
[2]Aix Marseille Univ, Inserm, INS, Institut de Neurosciences des Systèmes, Marseille, France
[3]Tokyo Tech World Research Hub Initiative (WRHI), Institute of Innovative Research, Tokyo Institute of Technology. Japan

April 15, 2020

1 Supporting Information

Recording	1	2	3	4	5	6	7	8	9
Number of neurons	67	41	31	34	73	58	33	52	33
Recording	10	11	12	13	14	15	16	17	18
Number of neurons	64	49	16	22	127	175	86	68	78

Table S1: Number of recorded neurons per recording.

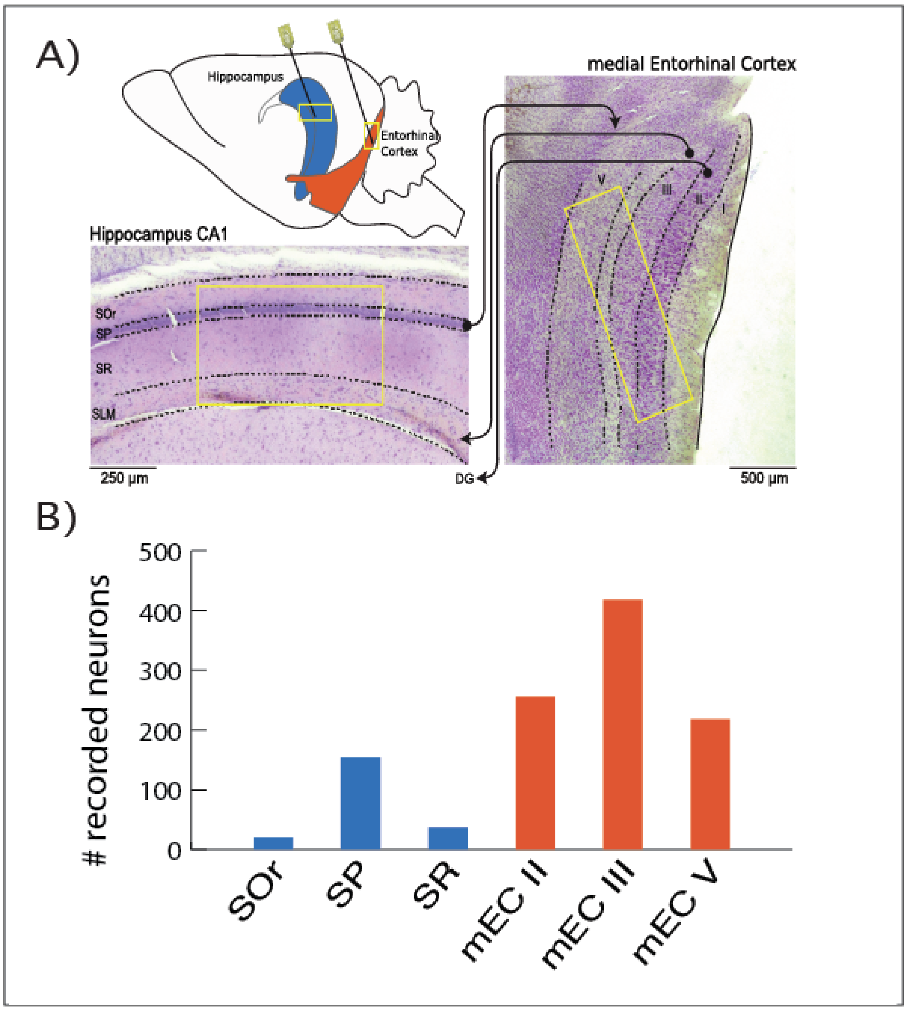

Figure S1: A) Simultaneous mEC/CA1 recording setup. **B)** Number of neurons recorded for each layer of each region: a majority of recorded neurons were located in the medial Entorhinal Cortex layers.

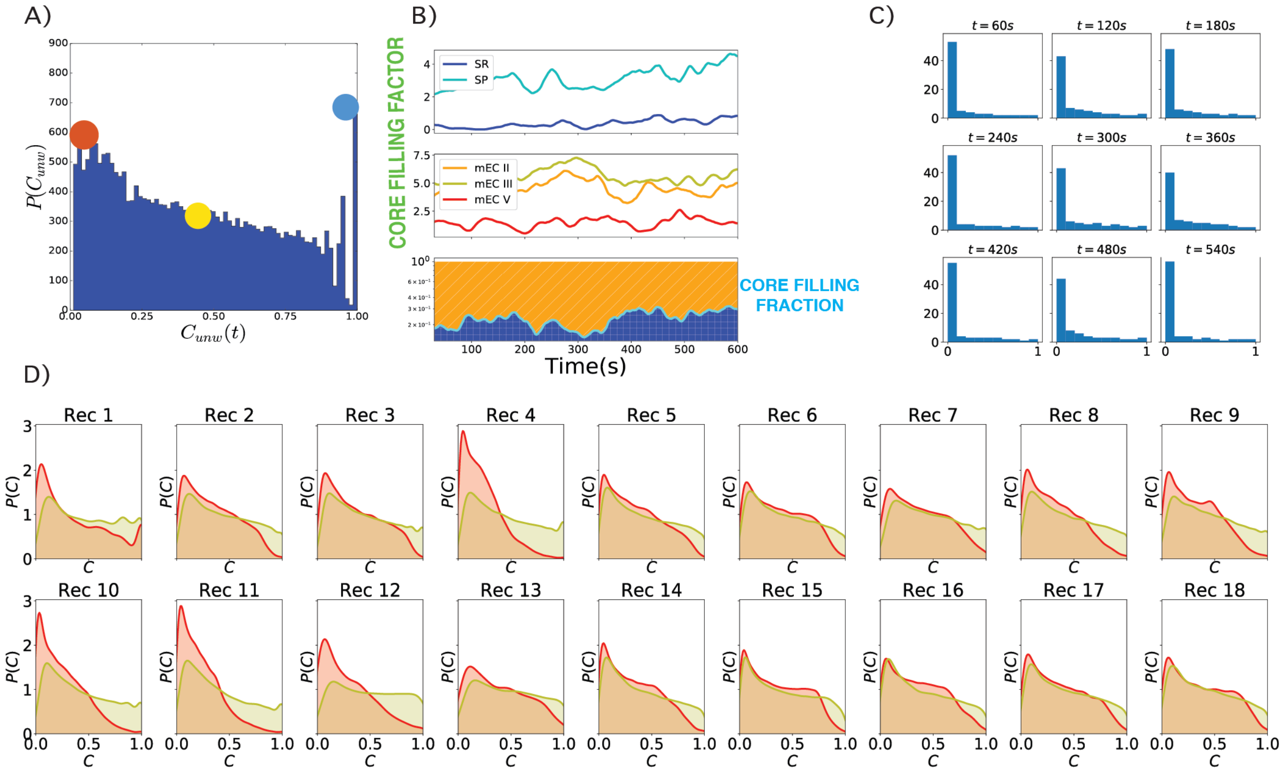

Figure S2: A) Histogram of the instantaneous unweighted coreness of all neurons in all time-steps for the same recording as in Figure 2 (unweighted analogous of Figure 2.A). **B)** Unweighted core-filling factors and core-filling fractions of the same recording as in Figure 2. **C)** Histograms of instantaneous unweighted coreness values for 9 different time-steps of the network's evolution. **D)** Density plots of the values of instantaneous weighted (red) and unweighted (yellow) coreness of all neurons at all times, separately for each recording.

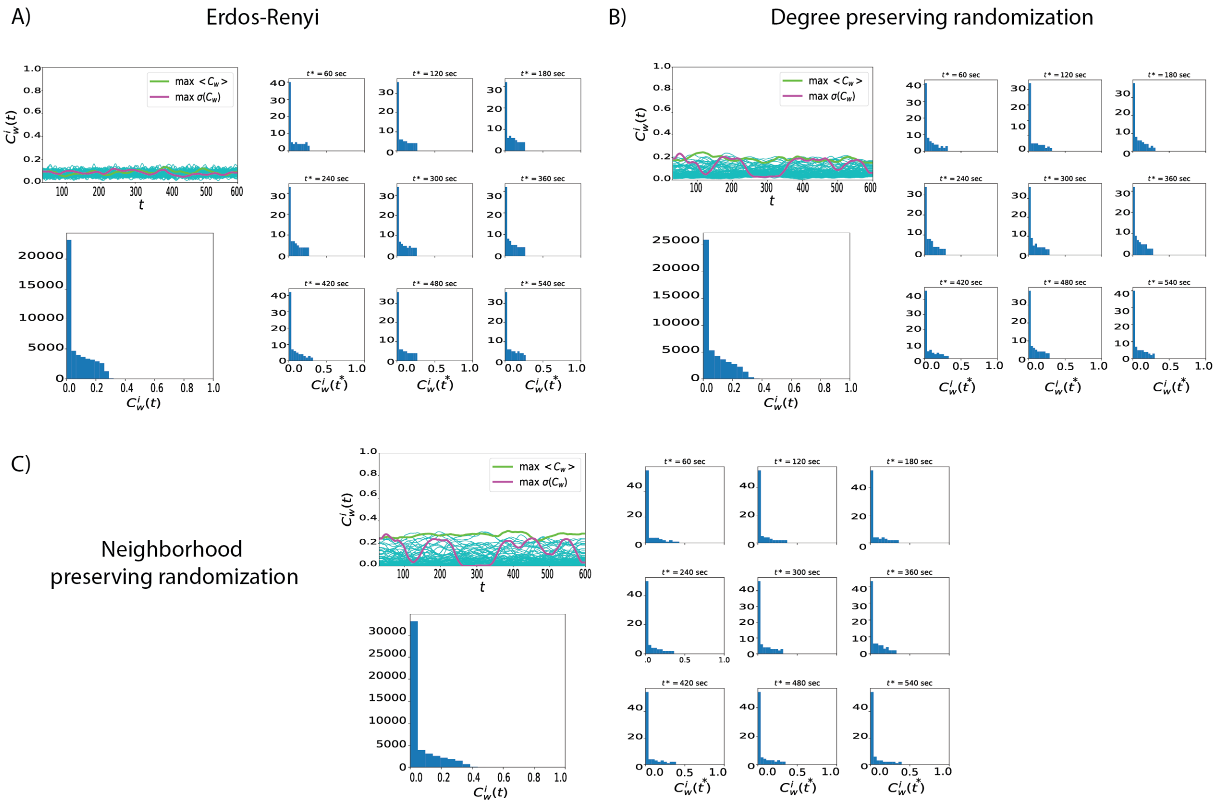

Figure S3: Here we analyze the time series of weighted instantaneous coreness of nodes and the distribution of these values for three null models. The first null model, in **A)**, is defined as a temporal network with at time t an Erdös-Rényi graph with the same number of nodes and edges as the experimental data at the same time t; from these plots we note how the coreness values of nodes display a noisy dynamics (left, above) and how their distributions for the total recording (left, below) and at different times (right) are peaked around low coreness values, with no high coreness values. The second null model, **B)**, is obtained by a randomization of the links at each time t, which preserves each node's degree Sneppen:2002; the plots of instantaneous coreness reveal patterns that somehow resemble those shown in Figure 2, however the coreness values are much lower than those measured for the experimental data. The third model, in **C)** corresponds to a randomization of the weights of the network's edges at each time, preserving the neighborhood (list of neighbors) of each node at each time; the instantaneous coreness plot shows different patterns for different nodes, as in the case of Figure 2, however the values of weighted coreness are sensibly lower.

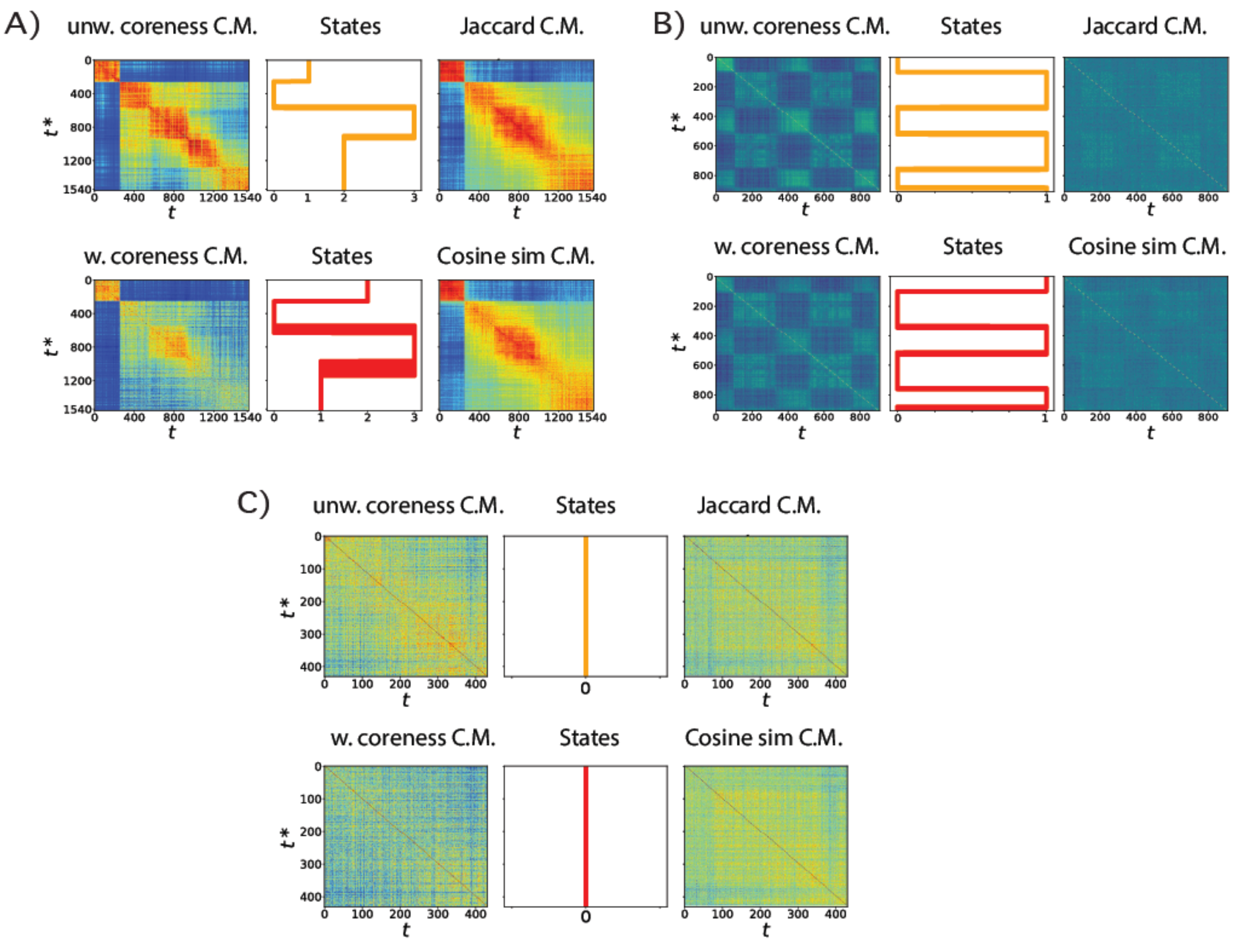

Figure S4: Recordings with different types of feature vectors correlation matrices and network states spectra. In each case, we show as in Figure 3.A. the temporal sequence of network states extracted by the unsupervised clustering of feature vectors, with on both sides the correlation matrices. Top plots correspond to unweighted features, bottom plots to weighted features. **A)** Case with diagonal blocks in the correlation matrices, with no off-diagonal blocks. The sequence of network states is in agreement with this structure, i.e. the network visits each network state only once. **B)** Case with chess-board-like correlation matrices. As seen from the network-state-spectra this recording is indeed in periodic oscillation between two states. **C)** Case in which no state can be clearly identified: this recording can be interpreted as in an extremely liquid single state, where both the core-periphery organization of the network and the neighborhoods of neurons change continuously with no clear temporal structure.

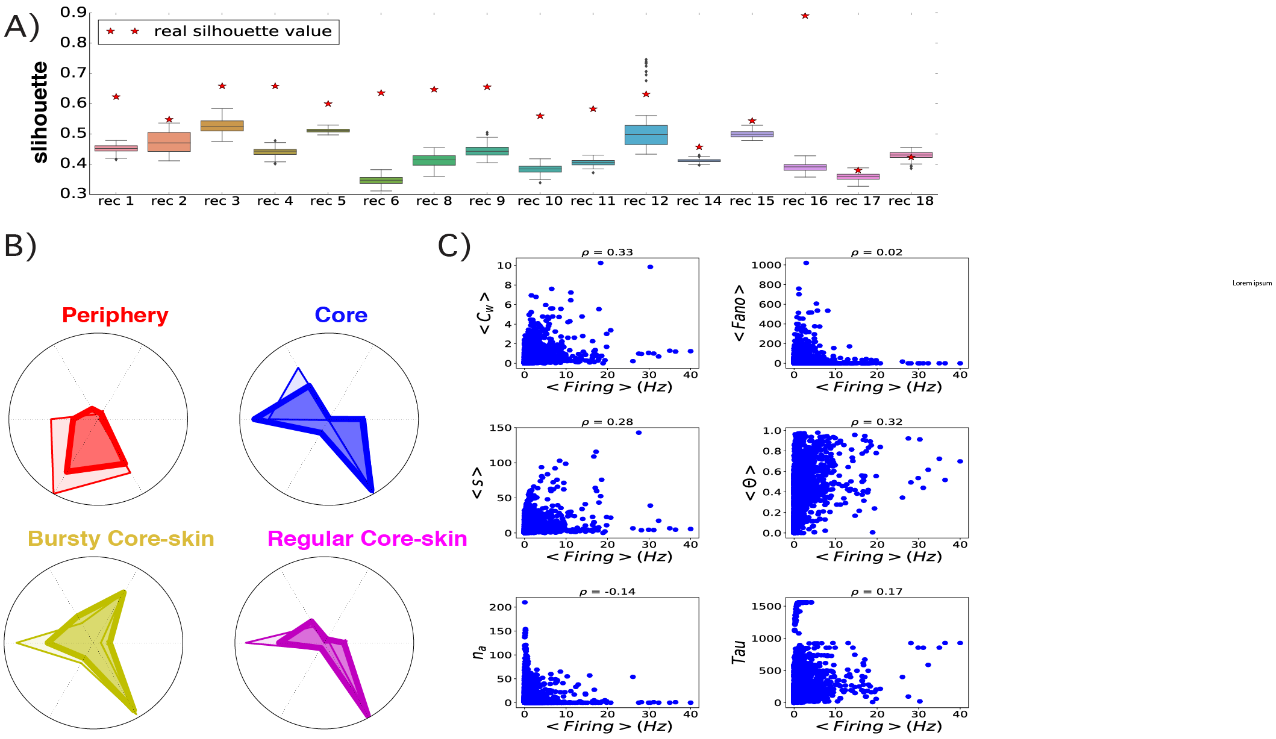

Figure S5: **A)** Silhouette plot: the red stars give for each recording the silhouette value for the Kmeans clustering performed on the connectivity profiles to retrieve the connectivity styles. These values are compared to distributions (boxplots) obtained for each recording by the following null model. We first reshuffle randomly the network-state labels of the time-frames of the whole recording while conserving the total length of each network-state. We then compute the connectivity profiles on the randomized states, cluster them in order to retrieve the connectivity styles, and compute the new silhouette. The boxplots correspond to the distribution of these null model silhouette values, obtained for 200 realizations of the reshuffling. The real silhouette values are well above the randomized distributions, suggesting that the definition of the discrete global states in the evolution of the information sharing liquidity and core-periphery organization is crucial for the analysis on the connectivity profiles and styles. **B)** Maximal connectivity profile of each connectivity style (lower opacity), as shown in Figure 4.C and connectivity profile of the centroid of the Kmeans clustering result (higher opacity). We stress that the centroid of the connectivity style does not correspond to the connectivity profile of any specific a neuron, but represents the average connectivity profile of the corresponding connectivity style. **C)** Scatterplots between each one of the 6 features computed for each neuron in each network-state of each recording and the average firing rate of the same neuron in the same network state. There is no evident relation between the average firing of a neuron in a network state and the network properties that we computed, showing that the various connectivity styles are not simply related to the neurons' activity.

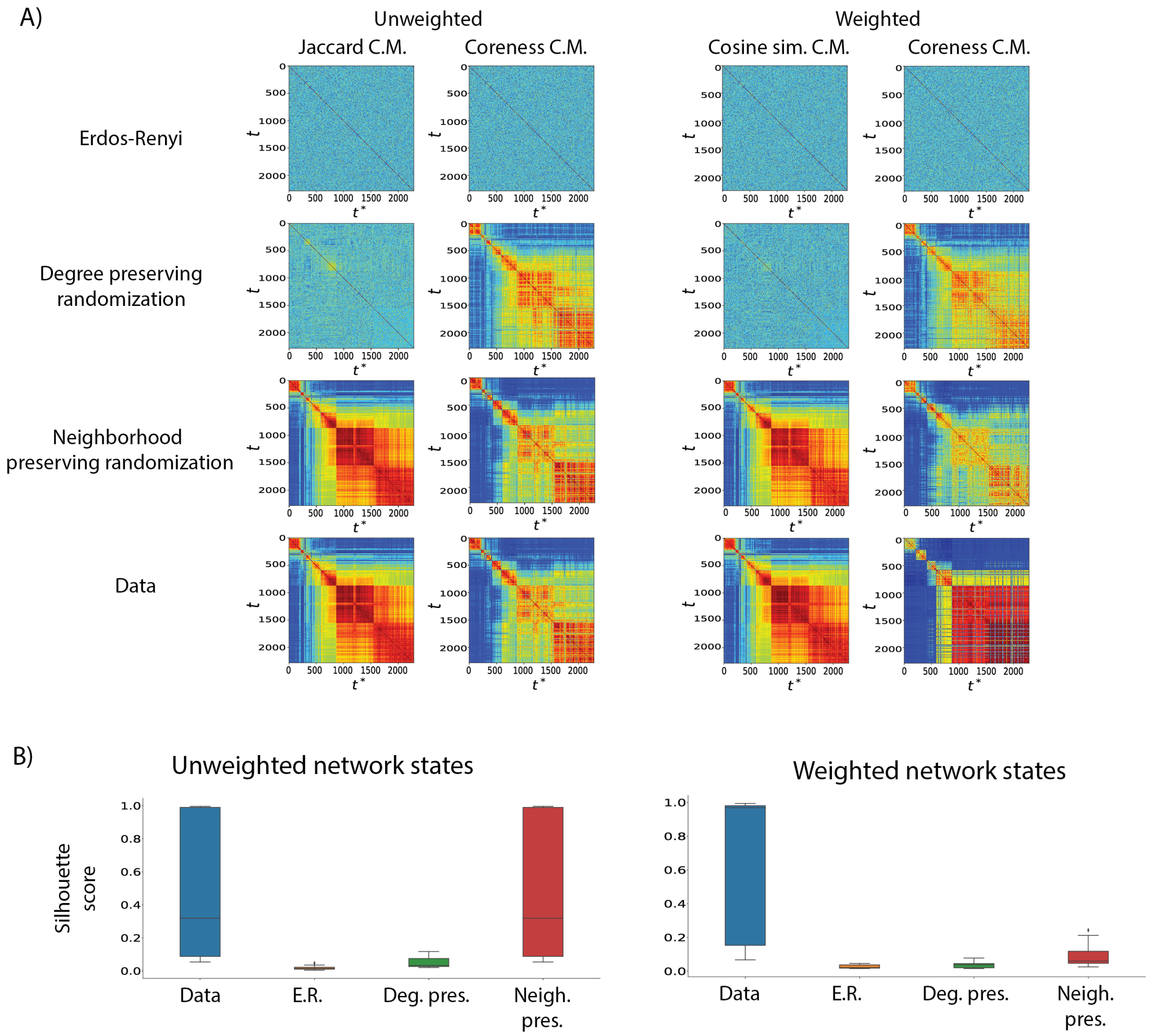

Figure S6: A) Each row shows four correlation matrices, two for the unweighted (left) and two for the weighted (right) liquidity and coreness feature vectors for a representative recording (the one used for Figure 3). Rows one to three correspond to the analysis carried out on the three different different null models (see Supporting Figure S3 and Methods) while the fourth row corresponds to the correlation matrices computed for the real data. The neighborhood preserving randomization leaves the unweighted structure of the network unchanged w.r.t. the experimental data (the unweighted correlation matrices for the experimental data and for the third null model are exactly the same). **B)** Distributions for all recordings of the average silhouette score for the K-Means clustering performed on the unweighted (left) and weighted (right) liquidity and coreness feature vectors time series to extract the network states spectrum for the temporal networks corresponding to the real data (blue) and the three null models. As the neighborhood preserving null model does not affect the unweighted structure of the networks, the networks states spectra extracted for this null model correspond exactly to the network states found for the unweighted feature vectors computed for the experimental data. The silhouette score values computed for the unweighted random and degree-preserving null models are sensibly lower compared to those found for the real data, and the distributions of silhouette score values found for all three weighted null models are all peaked around low values.

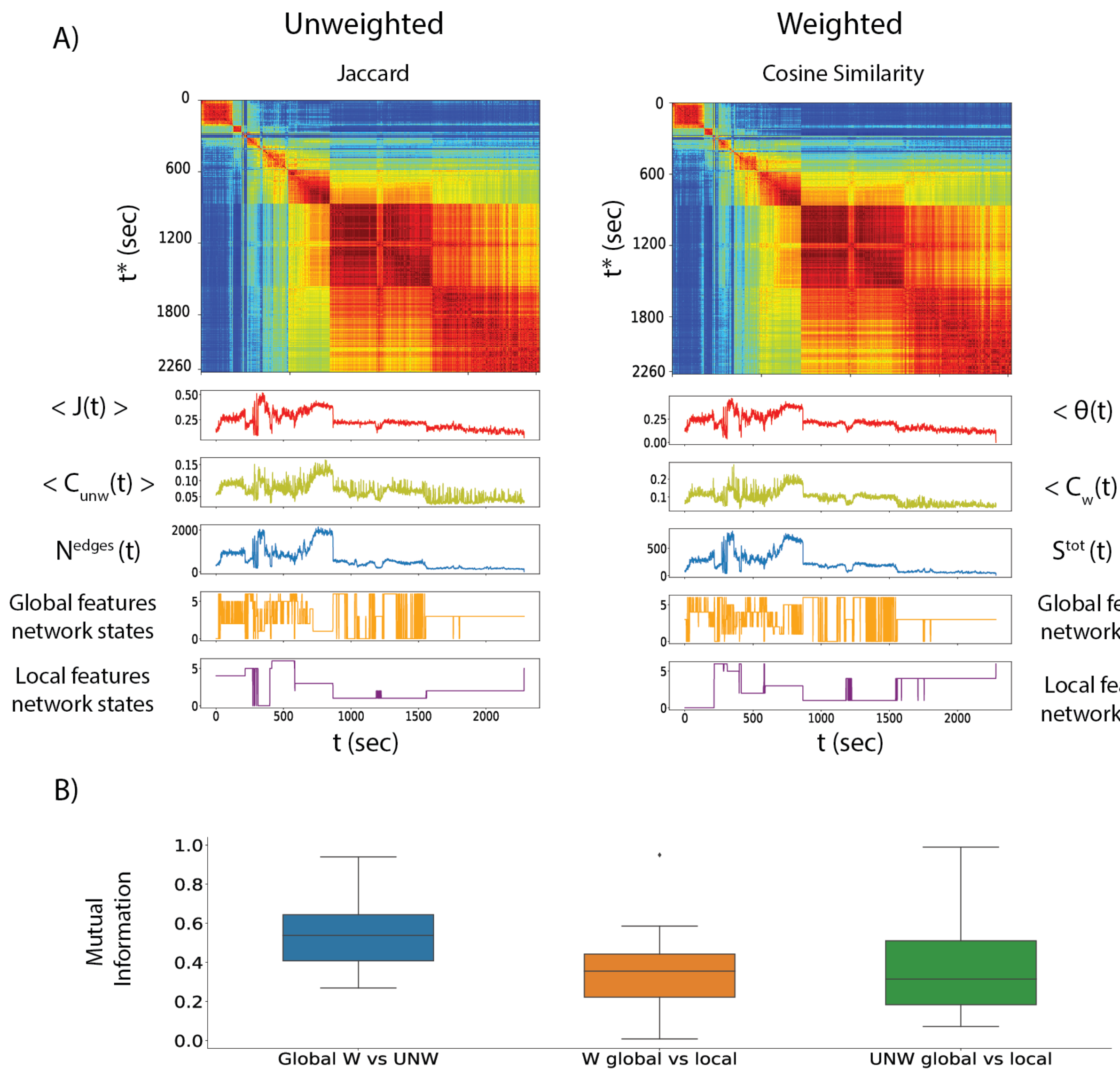

Figure S7: **A)** Top row: Global Jaccard index (left) and cosine similarity (right) matrices: the element $(t, t*)$ of each matrix represents the global Jaccard index (left) and cosine similarity (right) between the networks at time t and at time $t*$. Below, we plot in red the time evolution of the node-averaged Jaccard (left) and cosine similarity (right); the green curves give the node-averaged instantaneous unweighted (left) and weighted (right) coreness; in blue we plot the total number of edges of the network (left) and the total strength (right) at time t defined as $S^{tot}(t) = \sum_{i=1,\ldots,N} s^i(t)$ (see Methods for the definition of $s^i(t)$). The plot in orange displays the network states spectra obtained through a KMeans clustering on these three time series of global unweighted (left) and weighted (right) network features, the *global* network states; finally, in purple we plot the network states spectra computed for the local network features (as presented in Figure 3), i.e. the *local* network states. **B)** Boxplots of the distributions across all recordings of the amount of mutual information (see Methods) computed between: the unweighted and weighted global network states (in blue); the weighted global network states spectrum and the local network state spectrum (orange); the unweighted global network states spectrum and the unweighted local network states spectrum (green).

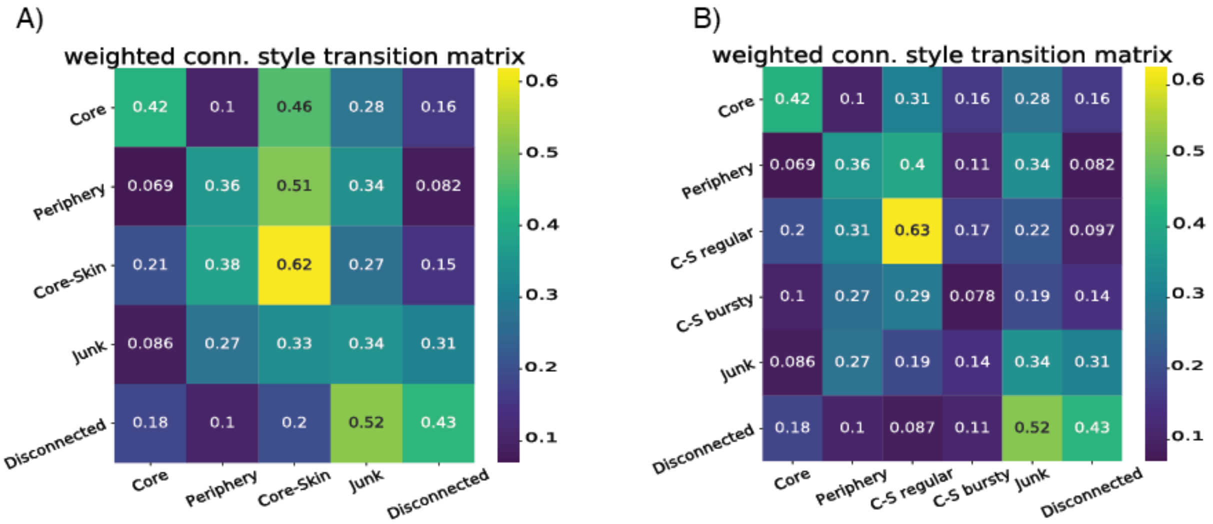

Figure S8: A) Transition matrix T_{ij} between connectivity styles of a neuron in successive network states taking into account the core, periphery, core-skin and junk connectivity styles, as well as network states in which the neurons are not connected to the rest of the network. **B)** Transition matrix T_{ij} between connectivity styles of a neuron in successive network states, taking into account the core, periphery, core-skin regular, core-skin bursty and junk connectivity styles, as well as the disconnected state of neurons. For both matrices, each diagonal element $T(i,i)$ represents the persistency rate of the corresponding connectivity style, i.e., the probability of a neuron to exhibit the same connectivity style in two successive global network states. The non-diagonal matrix elements are normalized on each row so that $\sum_{j\neq i} T(i,j) = 1$.

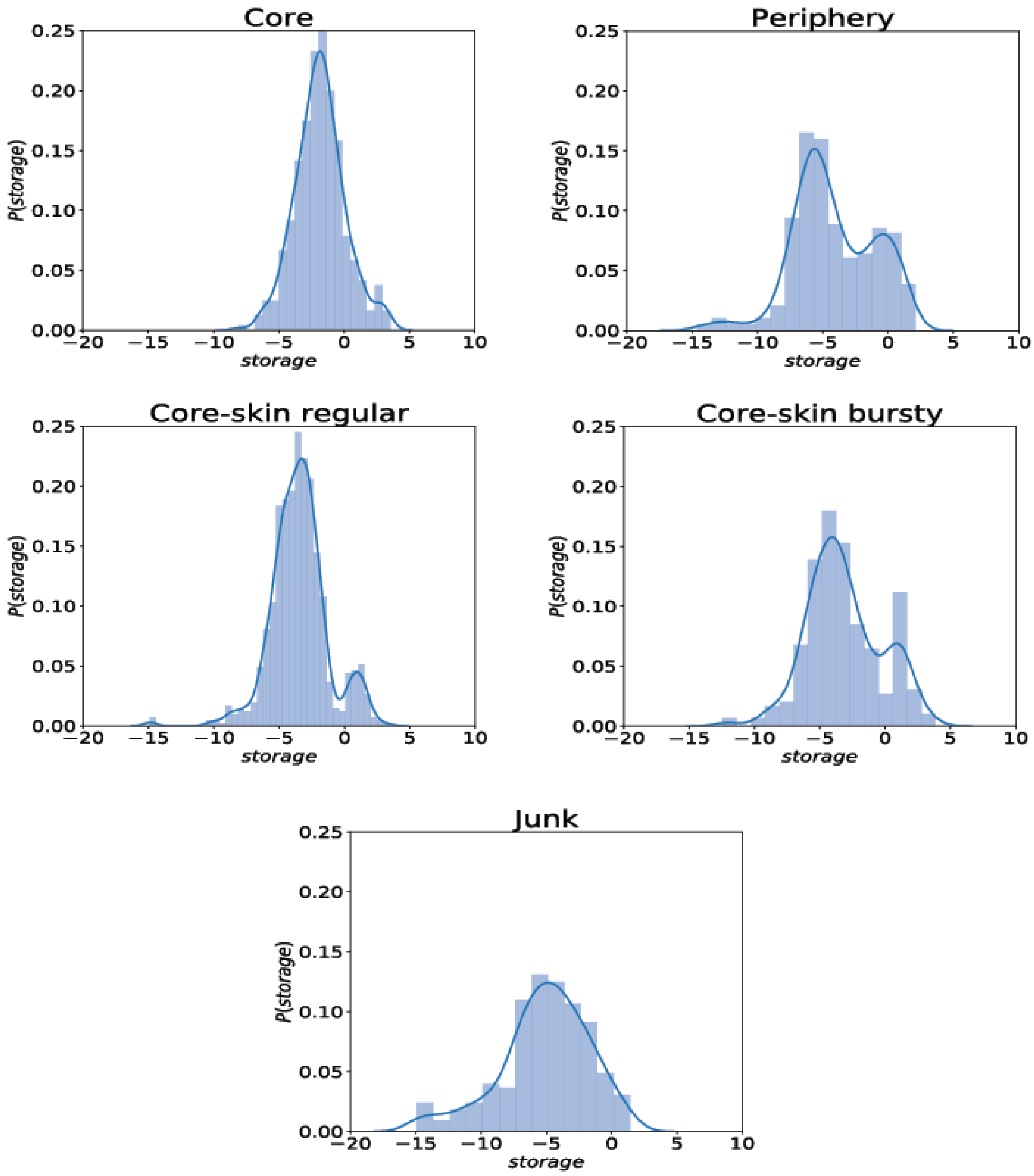

Figure S9: Density plots of the logarithm of the network-state-aggregated storage of connectivity profiles of each of the five (junk included) connectivity styles.

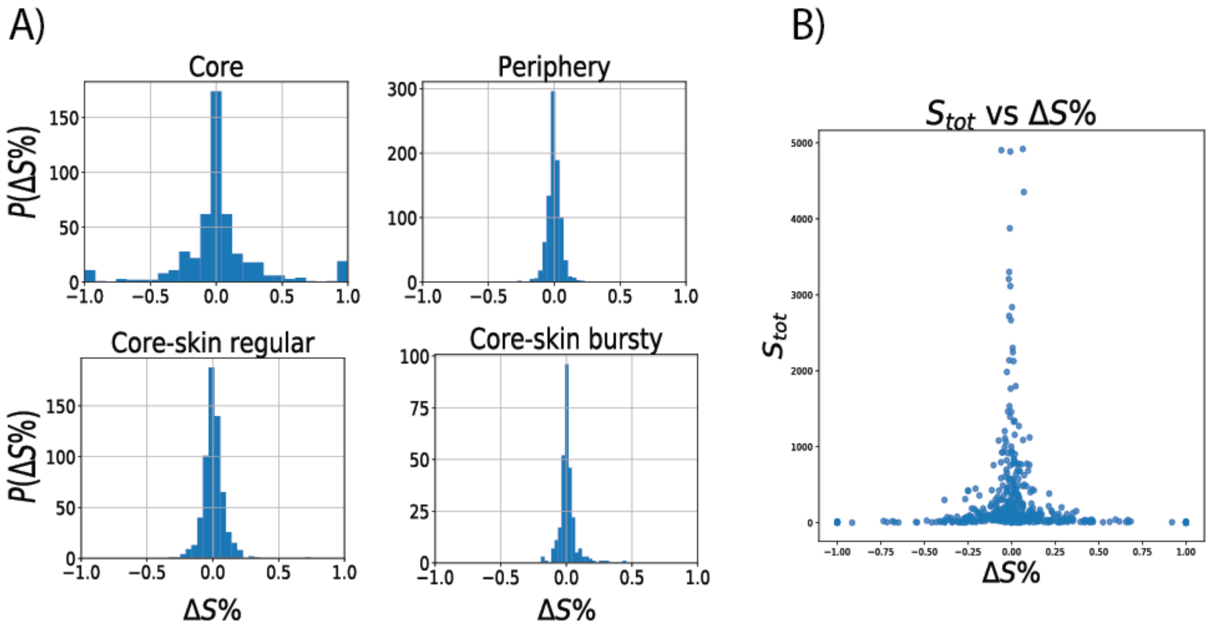

Figure S10: A) Distribution of values of $\Delta S\%$ for the connectivity profiles of each connectivity style. All the distributions of are peaked around 0, with the extreme values ($+1$ and -1) reached only for *core* connectivity profiles. The corresponding population of perfect senders and receivers represents a small portion of the overall number of connectivity profiles. **B)** Scatterplot of the values of S_{tot} vs. $\Delta S\%$, the former representing the total aggregate strength of a neuron during a state: $S^{i,h}_{tot} = s^{i,h}_{in} + s^{i,h}_{out}$. The scatterplot shows that perfect sender/receiver neurons ($\Delta S\% = \pm 1$) correspond to very low values of S_{tot}, hence they correspond to very weakly connected neurons.
